# The effect of damage to the white matter network and premorbid intellectual ability on postoperative verbal short-term memory and functional outcome in patients with brain lesions

**DOI:** 10.1371/journal.pone.0280580

**Published:** 2023-01-20

**Authors:** Kota Ebina, Mie Matsui, Masashi Kinoshita, Daisuke Saito, Mitsutoshi Nakada

**Affiliations:** 1 Laboratory of Clinical Cognitive Neuroscience, Graduate School of Medical Sciences, Kanazawa University, Kanazawa, Japan; 2 Laboratory of Clinical Cognitive Neuroscience, Institute of Liberal Arts and Science, Kanazawa University, Kanazawa, Japan; 3 Department of Neurosurgery, Kanazawa University, Kanazawa, Japan; 4 Department of Psychology, Yasuda Women’s University, Hiroshima, Japan; The University of Edinburgh, UNITED KINGDOM

## Abstract

Cognitive reserve is the capacity to cope with cognitive decline due to brain damage caused by neurological diseases. Premorbid IQ has been investigated as a proxy for cognitive reserve. To date, no study has focused on the effects of premorbid IQ in patients with brain tumors, considering the damage to white matter tracts. We investigated whether a higher premorbid IQ has a beneficial impact on postoperative verbal short-term memory and functional outcomes in patients with brain tumors. A total of 65 patients with brain tumors (35 right and 30 left hemisphere lesions) and 65 healthy subjects participated in the study. We used multiple regression analysis to examine whether white matter tract damage and premorbid IQ affect postoperative verbal short-term memory, and the interaction effects of premorbid IQ with damage to white matter tract on postoperative verbal short-term memory. Path analysis was used to investigate the relationship between damage to the white matter tract and premorbid IQ on postoperative functional ability. Our results showed that damage to the left arcuate fasciculus affected postoperative functional ability through verbal short-term memory, working memory, and global cognition in patients with left hemisphere lesions. In the right hemisphere lesion group, high premorbid IQ had a positive effect on functional ability by mediating verbal short-term memory, verbal working memory, and global cognition. We found that damage to the eloquent pathway affected postoperative verbal short-term memory regardless of the premorbid IQ level. However, a higher premorbid IQ was associated with better postoperative verbal short-term memory and functional outcomes when the brain lesions were not located in a crucial pathway. Our findings suggest that premorbid IQ and damage to the white matter tracts should be considered predictors of postoperative functional outcomes.

## Introduction

The level of cognitive impairment expressed as clinical symptoms does not always correspond to the severity of the brain pathology [1]. Cognitive reserve has been proposed to explain individual differences in cognitive resistance to dementia and cognitive decline or the discrepancy between the neuropathology of the brain and the expressed clinical symptoms [2–4]. Cognitive reserve refers to attempts to use existing cognitive processes or induce compensatory processes in undamaged areas to cope with brain damage, in contrast brain reserve refers to individual variations in brain characteristics, including brain size or number of neurons [3–5].

Brain reserve and cognitive reserve are associated with the risk of dementia. Regarding brain reserve, several studies have reported that brain size and head circumference as an indirect measure of premorbid brain size (brain reserve capacity) are associated with risk of Alzheimer’s disease [6, 7]. Although, head circumference is related to brain size measured using magnetic resonance imaging (MRI) [8, 9], head circumference does not necessarily represent an accurate measurement of brain size because it includes individual differences in skull thickness. Recent studies have used neuroimaging techniques to measure gray matter volume or white matter integrity as indicators of brain reserve [10]. The beneficial effects of cognitive reserve have been extensively investigated in patients with dementia. Studies have reported that higher educational levels, a history of higher occupational status, engagement in mentally stimulating leisure activities, and higher pre-dementia intellectual ability are associated with lower risks of dementia [11–18]. A recent meta-analysis of prospective cohort studies showed that higher cognitive reserve was associated with a 47% reduced relative risk of dementia or mild cognitive impairment [19]. The beneficial effects of cognitive reserve on cognitive decline and development of dementia are supported.

The effect of cognitive reserve has been extensively studied in patients with Alzheimer’s disease and the elderly population. Recently, attention has been paid to the beneficial effects of cognitive reserve in patients with brain lesions such as stroke, traumatic brain injuries, and brain tumors. Nunnari et al. [20] showed that higher premorbid intellectual ability and life exposure, such as education and leisure activities, were associated with functional outcomes in patients with stroke and traumatic brain injury. Umarova et al. [21] showed that years of education correlated with post-stroke cognitive function, including alertness, global cognition, working memory, and executive function. Furthermore, higher education levels were related to post-stroke better functional outcomes in acute stroke [21]. MacPherson et al. [22] found that education and premorbid IQ as a proxy of cognitive reserve predicted executive function and language function in patients with focal frontal lesions due to stroke or brain tumors. They showed that premorbid IQ predicts cognitive performance, such as verbal fluency, naming, and intelligence, following frontal and non-frontal lesions [23].

Brain damage from brain tumor infiltration is slower than that caused by stroke and traumatic brain injury. Low-grade gliomas exhibit brain reorganization of neural networks due to their slow infiltration [24–27]. Therefore, extensive studies have been conducted to elucidate the mechanisms of brain neuroplasticity in patients with brain tumors. Focusing on cognitive reserve in patients with brain tumors is effective in clarifying the neural basis of the cognitive reserve effect, considering that the beneficial effects of cognitive reserve on brain damage may be closely related to neuroplasticity.

A previous study suggested that a higher level of education is associated with better neuropsychological rehabilitation-related improvement and general improvement [28]. Campanella et al. [29] showed that premorbid IQ was the best predictor of preoperative language function computed from the performance of the phonemic fluency task, token test, picture naming task, auditory repetition task, writing, and reading task. Although some studies have focused on cognitive reserve in patients with brain tumors, the contribution of cognitive reserve to brain function has been supported.

Maximal resection is well known as an effective treatment that contributes to overall survival [30]. However, damage to the brain, such as the cerebral cortex and white matter tracts after resection, affects higher brain function related to patients’ quality of life. In the treatment of brain tumors, it is important to consider the balance between the prognosis of function and survival [31, 32]. Mapping language eloquent areas by awake surgery is important to preserve language function, as language function is an integral part of daily life and the language network is more extensive and complex. Previous studies have identified a relationship between language function and white matter tracts aimed at avoiding permanent language deficits [33, 34].

Clinical research in patients with brain lesions primarily uses two different approaches to measure the damage to specific white matter tracts. The first approach directly measures the integrity of white matter connections using diffusion-weighted imaging (DWI). The second approach indirectly measures the damage to white matter tracts by overlaying a patient’s lesion map on an atlas of white matter tracts created from DWI datasets obtained from healthy controls [35]. This indirect approach estimates the probability and proportion of disconnection for a specific white matter tract based on the number of voxels overlapping the lesion map on the atlas of healthy white matter tracts. The direct approach using DWI data is more reliable for measuring damage to the white matter in patients with brain lesions. However, DWI is difficult to perform in clinical situations, especially in patients who have difficulty undergoing MRI for longer periods of time. Therefore, the indirect approach is widely used in studies examining the association between white matter damage and cognitive function in patients with brain lesions. The results of the indirect approach are reportedly consistent with those of the direct approach. For example, Thiebaut et al., [36] estimated the pattern of disconnection of white matter tracts given by the lesions using a diffusion tensor imaging (DTI) atlas previously published by them [37]. They showed that spatial neglect is associated with disconnection of superior longitudinal fasciculus (SLF) Ⅱ measured using this indirect approach, and that this result is supported by individual white matter tractography dissection [36]. Pacella et al., [38] also depicted a patient’s lesion as a region of interest (ROI), which they overlaid an atlas of white matter tracts created from healthy DWI data to measure the disconnection of the white matter tract of the ROI using Tractotron in the BCB toolkit [35]. The disconnections of white matter tracts such as the SLF, arcuate fasciculus (AF), and inferior longitudinal fasciculus indicated by the indirect approach were also confirmed in subsequent direct analysis using DTI [38]. Furthermore, Wawrzyniak et al., [39] successfully confirmed the well-known relationship between damage to white matter tracts and symptoms caused by disconnection of specific white matter tracts using voxel-wise disconnection mapping as an indirect approach. They showed the validity and utility of the methodology of the disconnectome maps, an indirect approach to investigate the relationship between white matter tract disconnection and cognitive impairment using an atlas obtained from healthy controls [39].

The arcuate fasciculus (AF) is a white matter tract that is classically believed to be involved in language function and connects the inferior frontal gyrus (Broca’s area) and the superior temporal gyrus (Wernicke’s area) [40–42]. Catani et al. [43] showed that anatomic AF consists of anterior, long, and posterior segments and suggested that the long segment of AF (direct pathway) involves the phonological process that is the basis of the language and that the anterior and posterior segments of AF (indirect pathway) involve the lexical-semantic process. Previous studies have shown that AF is related to language functions, including repetition, naming, comprehension, fluency, and verbal short-term memory (VSTM) [44–47].

Furthermore, VSTM is a key factor in language processing that involves AF and is part of the working memory system that is related to daily living functions such as language comprehension, mental arithmetic, and learning [48]. The phonological loop in the most common working memory model of this function distinguishes between a phonological store, which holds a memory trace for 1 to 2 seconds, and the articulatory process, which can refresh the memory trace [48, 49]. For the neural basis of VSTM, the angular gyrus, supramarginal gyrus, and pars opercularis have been indicated to be involved in the left hemisphere [50–53]. Furthermore, a previous study using direct electrical stimulation (DES) showed that DES of the AF anterior segment elicited the VSTM impairment [47]. Therefore, these findings suggest that AF is a crucial white matter tract for VSTM as a working memory system, and damage to the AF is likely to affect VSTM and working memory impairment.

Few studies have focused on the effects of cognitive reserve on cognitive impairment in patients with brain lesions. Although Campanella et al. [29] investigated the relationship between language function and premorbid intellectual ability (as a proxy for cognitive reserve), no study has examined the relationship between postoperative language function and premorbid intellectual ability, considering damage to white matter tracts involving language function in patients with brain tumors. Clarifying the relationship between damage to white matter tracts and cognitive reserve, and the effects on postoperative cognitive function may help predict postoperative cognitive performance more accurately. Furthermore, quality of life (QOL) or daily living function assessments such as the Karnofsky Performance Status (KPS) are key postoperative functional outcomes. It is clinically important to determine whether cognitive reserve affects functional outcomes at the level of daily living as well as on neuropsychological performance such as VSTM and working memory in patients with brain tumors.

This study primarily aimed to identify the impacts of premorbid intellectual ability (as a proxy of cognitive reserve) and damage to white matter tracts on postoperative VSTM and functional outcomes relating to daily life. Therefore, we investigated whether damage to the left AF white matter tract and premorbid IQ are associated with postoperative VSTM and daily living functionality. We hypothesized that damage to AF negatively affects postoperative VSTM, while premorbid intellectual ability positively affects postoperative VSTM, and that these factors associated with VSTM directly or indirectly affect functional ability through VSTM, working memory, and global cognition.

We compared neurocognitive performance between patients and healthy age-matched controls to determine the severity of cognitive impairments such as VSTM, working memory, picture naming, and verbal fluency. We analyzed the data for patients with left and right hemispheric lesions separately because patients with right hemispheric tumors do not have left AF damage. We used partial correlation analyses to explore the impacts of white matter tract disconnections and premorbid IQ on postoperative VSTM. It is important to clarify whether the postoperative VSTM can be better explained by including variables of cognitive reserve in addition to white matter tract damage. We performed hierarchical multiple regression analyses to identify whether the explanatory power for postoperative VSTM is significantly increased by this inclusion of premorbid IQ. Additionally, we investigated whether cognitive reserve moderates the association between damage to white matter tracts such as the AF, and postoperative VSTM performance, via multiple regression analyses.

Path analysis was used to comprehensively investigate the relationship between damage to white matter tracts and premorbid IQ on postoperative functional outcomes, such as that assessed by the KPS. When investigating whether cognitive reserve affects KPS, we considered two patterns: (1) the direct effect of premorbid IQ on KPS, and (2) the indirect effect of premorbid IQ on KPS as mediated by cognitive performance such as VSTM. Identifying both the direct and indirect impact of premorbid IQ on KPS will help comprehensively elucidate the relationship between premorbid IQ, cognitive function, and daily function.

Although VSTM is one of the basic neurocognitive functions involved in daily living, it is not always possible to accurately predict postoperative daily living functionality by VSTM performance alone. This is because various cognitive functions affect postoperative daily functionality, such as working memory and global cognitive state. VSTM is a component of the working memory system, which is a crucial function in global cognition. Thus, when investigating the association between premorbid IQ and postoperative daily function, we considered VSTM as well as working memory relating to VSTM and global cognition relating to working memory in the path analysis. As for the relationships between variables of cognitive function such as VSTM, working memory, and global cognition, we examined a model wherein VSTM affects working memory, working memory affects global cognition, and global cognition affects daily living functionality. The reasons for placing the variables for cognitive function in this order in the path analysis is that high-level cognitive processing is based on more basic levels of cognitive processing. An inverse relationship is not appropriate, for example, a path direction in which working memory or global cognitive performance affect VSTM. Therefore, it is appropriate to model the order in which the more basic VSTM impacts working memory performance, working memory influences global cognitive status (which involves VSTM and working memory), and global cognitive status (which covers a broader cognitive domain) impacts functional outcomes of daily life.

## Materials and methods

### Procedures

We recruited the patients from the Department of Neurosurgery at Kanazawa University Hospital through their doctors. All patients underwent brain tumor resection at the Kanazawa University Hospital. We measured premorbid IQ, global cognitive state as a screening test, daily living skills, and neuropsychological performance for verbal fluency, VSTM, and verbal working memory in patients with brain tumor whose clinical condition was stable at least 1 month after surgery. MRI scans were obtained at least 1 month after surgery in clinically stable conditions.

We recruited healthy participants from Kanazawa University by displaying their posters. Sixty-five healthy participants, as the control group, received the assessment of premorbid IQ (estimated IQ) and neuropsychological tests for verbal fluency, VSTM, and verbal working memory. We did not conduct assessments of global cognitive state as a screening test and daily living skills in healthy participants since we recruited healthy participants without neurological illness, psychiatric disease, or a history of head trauma or surgery, as described in the following exclusion criteria for healthy controls. Hand preference was rated using the Japanese hand preference questionnaire for participants and healthy controls [54].

### Participants

Sixty-five patients with tumors located within the frontal lobe (N = 29), temporal lobe (N = 20), parietal lobe (N = 9), occipital lobe (N = 4), and others (N = 3) were included in this study (35 right and 30 left hemisphere lesions). Thirty-one patients underwent radiotherapy. Sixty-five age-matched healthy participants were also included in the study.

**Fig 1:**
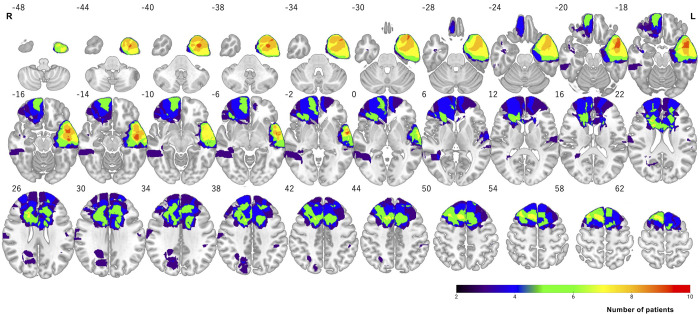
Overlay map of the resection cavity in patients with brain lesions. Maximum resection cavity overlaps in the left temporal lobe. Red indicates an area with a large overlap of the resection cavities. R, Right; L, Left.

To determine the location of the tumor resection cavity, we created an overlay map of the resection cavities by overlaying the resection cavity data for each patient using MRIcron (Fig 1). In patients with right hemisphere lesions, the tumor resection cavities overlapped in the corpus callosum, supplementary motor area, and middle frontal gyrus. Most patients with left hemisphere brain lesions had resection cavities located in the inferior temporal gyrus, middle temporal gyrus, and superior temporal gyrus.

These patients were diagnosed with brain tumors such as oligodendroglioma (n = 11), anaplastic astrocytoma or oligodendroglioma (n = 14), diffuse astrocytoma (n = 9), glioblastoma (n = 18), metastatic brain tumor (n = 3), dysembryoplastic neuroepithelial tumor (n = 1), ependymoma (N = 2), cavernous angioma (n = 1), ganglioglioma (N = 1), glioneuronal tumor (N = 1), hemangioblastoma (N = 1), meningioma (N = 1), pleomorphic xanthoastrocytoma (N = 1), and schwannoma (N = 1).

Patients with a premorbid IQ less than 70 were excluded. The exclusion criteria for healthy participants were as follows: (a) participants with a history of traumatic head injury and surgery, (b) participants with neurological illness, and (c) participants with alcohol or substance abuse. The demographic characteristics of the participants are presented in Table 1. This study was approved by the medical ethics committee at Kanazawa University [No. 2018–140 (2897)], and written informed consent was obtained from all participants after the procedures were fully described to them. This study was conducted following the guidelines of the Internal Review Board of Kanazawa University.

**Table 1:** Means and standard deviations of evaluations.

Group	Healthy (N = 65)	Patient (N = 65)	t value	P value	Left lesion (N = 30)	Right lesion (N = 35)	t value	P value
	Means (SD)				Means (SD)			
Age (years)	50.1 (13.3)	52.5 (14.9)	t = -0.97	p = 0.33	49.1 (14.0)	55.5 (15.3)	t = -1.77	p = 0.08
Education (years)	15.5 (2.7)	13.5 (1.9)	t = 4.86	p < 0.001	13.5 (1.9)	13.5 (2.0)	t = 0.01	p = 0.99
JART	105.4 (7.9)	98.4 (11.5)	t = 3.95	p < 0.001	97.1 (10.3)	99.5 (12.5)	t = -0.79	p = 0.43
Digit span forward	6.5 (1.3)	6.0 (1.4)	t = 1.74	p = 0.08	6.1 (1.4)	6.0 (1.3)	t = 0.38	p = 0.71
Digit span backward	5.1 (1.4)	4.0 (1.0)	t = 5.17	p < 0.001	3.9 (1.2)	4.1 (0.9)	t = -0.74	p = 0.46
VFT “ka”	13.4 (3.9)	9.6 (4.8)	t = 4.82	p < 0.001	8.2 (4.3)	10.8 (4.9)	t = -2.05	p = 0.05
VFT ANIMAL	18.2 (4.1)	13.6 (6.0)	t = 4.86	p < 0.001	12.2 (6.8)	14.8 (5.1)	t = -1.71	p = 0.09
MMSE		26.8 (4.0)			26.1 (4.8)	27.3 (3.0)	t = -1.19	p = 0.24
KPS		90.5 (10.1)			89.7 (9.3)	91.1 (10.8)	t = -0.59	p = 0.56
Picture naming (%)		93.6 (17.2)			86.6 (23.6) 23.6 23.6 23.6	99.5 (1.9)	t = -2.67	p = 0.01

JART, Japanese Adult Reading Test; VFT, verbal fluency task; MMSE, Mini-Mental State Examination; KPS, Karnofsky Performance Status; Left lesion, left hemisphere lesion group; Right lesion, right hemisphere lesion group.

### Measures

#### Postoperative assessment of VSTM and working memory

The digit span task in forward and backward conditions was performed using a subtest of the Clinical Assessment for Attention [55]. The digit span forward task was used to measure VSTM, and the digit span backward task was performed to measure verbal working memory. For the digit span test, the number of digits that the participants achieved was scored. A higher number of achieved digits implies a higher short-term and working memory performance.

#### Language

We performed fluency and naming tasks that included the Western Aphasia Battery [56] as part of a subtest. The verbal fluency task (VFT) was administered to healthy subjects and patients, while the picture naming task was administered only to patients. In the VFT, participants were asked to generate as many words as possible within 60 s. We implemented two categories: words beginning with “ka” and ANIMALS. We used the total number of words generated in each category as the score. In the picture naming task, patients were asked to answer the names of a series of 60 objects. The number of correct answers to the names of the objects was scored.

#### Global cognition and functional ability

Global cognitive function was measured using the Mini-Mental State Examination (MMSE) [57]. Postoperative daily living function and independence levels (functional ability) were assessed using the Karnofsky performance status (KPS) for each patient.

#### Premorbid intellectual ability

We assessed premorbid intellectual ability using Japanese Adult Reading Test (JART) [58]. JART consists of 50 irregular Japanese words (kanji), which are ideographic scripts. Participants were required to read the words, and the premorbid full-scale IQ, verbal IQ, and performance IQ were evaluated based on the number of errors in reading performance. We used premorbid full-scale IQ as the premorbid IQ score.

### Magnetic resonance image

T1 MR images were acquired postoperatively using 3D T1-weighted sequences on a 3.0 Tesla MRI scanner (Signa Excite HDx 3.0T, General Electric Medical Systems). The T1 MR image parameters were as follows: repetition time, 8.8 ms, echo time, 3.5 ms; slice thickness,1.2 mm; and field of view, 256 × 256 mm.

### Disconnection analysis

The patients’ MRI images were normalized into the MNI (Montreal Neurological Institute) space using SPM 12 (Statistical Parametric Mapping; https://www.fil.ion.ucl.ac.uk/spm/software/spm12/) implemented in MATLAB (R2019a, version 9.6; The MathWorks, Inc). MRIcron software (https://people.cas.sc.edu/rorden/mricron/index.html) was used to reconstruct the resection cavities. All reconstruction resection cavities were first manually drawn by K. E. and subsequently checked by an experienced neurosurgeon (M. K.) The damage to the white matter tracts of patients was calculated using Tractotron analysis in the BCB toolkit (https://storage.googleapis.com/bcblabweb/index.html). Tractotron is an atlas-based approach that measures the probability of damage to each white matter tract at the individual level by overlaying a patient’s lesion map on an atlas of white matter tracts based on healthy controls published by Rojkova et al. [35, 59]. We therefore measured each patient’s probability of white matter tract disconnection indirectly using an atlas-based approach without diffusion images.

### Statistical analyses

#### Comparison of cognitive performance

A two-sample t-test was performed to compare cognitive performance between the healthy control and patient groups and between the right and left hemisphere lesion groups.

#### Association between white matter tract, cognitive reserve proxy and postoperative cognitive performance

We calculated the partial correlation coefficient to investigate whether the left AF correlates with the digit span forward and backward scores for the left hemisphere lesion groups and whether the disconnection of white matter tracts correlates with digit span forward and backward scores for the right hemisphere lesion groups, using age as a covariate. As for the left hemisphere lesion group, we also investigated the relationship between the left AF and language functions, such as VFT and picture naming. We investigated the association between KPS scores and the disconnection ratio of the white matter tract. In this analysis, white matter tracts with extremely small numbers of patients for whom disconnection rates were calculated were excluded from the analysis.

We performed hierarchical multiple regression analysis to investigate whether adding the cognitive reserve proxies as an independent variable would significantly increase the explanatory power of the regression model over a regression model that included damage to the white matter tract. Therefore, we used the stepwise method, which includes more highly associated independent variables in the regression model, rather than the forced entry method, which includes all independent variables in the regression model. However, in the step that included age as an independent variable, the forced entry method was used to consider age as a covariate in the regression model.

For the left hemisphere lesion group, in Step 1, age was entered as an independent variable (forced entry method); in Step 2, the disconnection ratio of the AF segments (anterior, long, and posterior) was entered as an independent variable (stepwise method); in Step 3, JART scores and years of education (as a proxy for cognitive reserve) were entered as independent variables (stepwise method). Additionally, we investigated whether our cognitive reserve proxy moderates the relationship between the disconnection of the AF segment and digit span forward score via multiple regression analyses with the forced entry method. This analysis included age, cognitive reserve proxy, disconnection of AF, and the interaction of cognitive reserve proxy with disconnection of the AF segment (cognitive reserve proxy × disconnection of AF) as independent variables.

For the right hemisphere lesion group, in Step 1, age was entered as an independent variable (forced entry method); in Step 2, the disconnection ratio of the right white matter tracts, which showed a significant correlation in the partial correlation analysis, was entered as an independent variable (stepwise method); and in Step 3, the JART scores and years of education (as a proxy for cognitive reserve) were entered as independent variables (stepwise method). Similar to the analysis in the left hemisphere lesion group, we examined the interaction effects of cognitive reserve and right white matter tract disconnection via multiple regression analyses with the forced entry method. This analysis included age, cognitive reserve proxy, disconnection of right white matter tract (which was shown to be associated with digit span forward score in a prior multiple regression analysis), and the interaction of cognitive reserve proxy with disconnection of the right white matter tract (cognitive reserve proxy × disconnection of right white matter tract) as independent variables.

#### The relationship between cognitive reserve proxy and functional abilities

Path analysis was performed to investigate whether premorbid IQ and damage to the white matter tracts affected KPS as postoperative functional ability. We investigated two patterns of the relationship between premorbid IQ and KPS: (1) the direct impact of premorbid IQ on KPS and (2) the indirect impact of premorbid IQ on KPS as mediated by cognitive performance such as VSTM, working memory, and global cognitive status. Regarding the left hemisphere lesion group, premorbid IQ and left AF segments that showed significant associations in the multiple regression analysis were included in the path analysis. For the right hemisphere lesion group, premorbid IQ and right white matter tracts that were associated with VSTM in multiple regression analysis were included in the path analysis. Age was entered into the model as a covariate because it affects cognitive function. Path analysis was performed separately for the right hemisphere lesion and left hemisphere lesion groups. We performed path analysis using IBM SPSS AMOS, version 27.0 (Armonk, NY, IBM Corp.).

## Results

### Demographics and cognitive data

Table 1 shows the demographic and cognitive data of patients and healthy controls. For healthy controls, the mean age of the participants was 50.1 ± 13.3 (standard deviation, SD) years, and the mean years of education was 15.5 ± 2.7. Fifty-seven participants were right-handed, four participants were left-handed, and four participants were both-handed. The mean JART score was 105.4 ± 7.9 (SD).

For patients, the mean of patients’ age, years of education, and JART score was 52.5 ± 14.9 (SD) years, 13.5 ± 1.9, and 98.4 ± 11.5 (SD), respectively. Forty-two patients were right-handed, four were left-handed, 14 were both-handed, and five patients had not been evaluated. The mean picture naming score was 93.6 ± 17.2 (SD). The mean of MMSE score and KPS score was 26.8 ± 4.0 and 90.5 ± 10.1, respectively. More details are provided in Table 1.

The distribution of each variable was assessed comprehensively using the Kolmogorov–Smirnov test, assessments of skewness and kurtosis, and Q–Q charts. Most variables were normally distributed; however, the patients’ picture-naming task scores suggested non-normality.

### Comparison of cognitive performance

Although normality was not confirmed for the picture-naming task, all tests in this study were unified into t-tests. Mann-Whitney tests were performed on this variable, providing results similar to those of the t-test. The results of two sample t-test showed that patients with brain lesions were more impaired than the healthy control in the digit span backward (mean, 4.0 ± 1.0 vs. 5.1 ± 1.4, t = 5.17, p < 0.001), VFT “ka” (mean, 9.6 ± 4.8 vs. 13.4 ± 3.9, t = 4.82, p < 0.001), VFT ANIMAL (mean, 13.6 ± 6.0 vs 18.2 ± 4.1, t = 4.86, p < 0.001). There were no significant differences in the digit span forward scores between the patient and healthy groups.

Regarding comparison between the left hemisphere lesion and right hemisphere lesion groups, the results of the t-test showed that the left hemisphere lesion group was more impaired than the right hemisphere lesion group in the picture naming scores (mean, 86.6 ± 23.6 vs. 99.5 ± 1.9, t = -2.67, p = 0.01). There were no significant differences between the left hemisphere lesion and right hemisphere lesion groups in the digit span forward, digit span backward, VFT “ka,” and VFT ANIMAL. More details are provided in Table 1.

### Correlation between the white matter tract and postoperative cognitive performance

The results of the partial correlation coefficients showed that the digit span forward scores were significantly negatively associated with the disconnection ratio of the left AF anterior segment (r = -0.44, p = 0.02), left AF long segment (r = -0.43, p = 0.02), left AF posterior segment (r = -0.45, p = 0.02) in the left hemisphere lesion group. We also found that digit span backward scores were significantly correlated with the disconnection ratio of the left AF long segment (r = -0.50, p = 0.01) in the left hemisphere lesion group. The disconnection ratio of any segment of left AF did not correlate with VFT and picture naming. For the right hemisphere lesion group, the digit span forward scores were significantly correlated with the disconnection ratio of the right cingulum posterior (r = - 0.46, p = 0.01), whereas the disconnection ratio of the white matter tracts was not correlated with the digit span backward scores.

Regarding the relationship between KPS scores and the white matter tracts, KPS score was significantly associated with the left anterior commissure (r = -0.45, p = 0.02), left AF anterior segment (r = -0.41, p = 0.03), left frontal insular tract 3 (r = -0.55, p = 0.002), left frontal insular tract 4 (r = -0.45, p = 0.02), left frontal insular tract 5 (r = -0.37, p = 0.05), left inferior longitudinal (r = -0.49, p = 0.006), and left optic radiation (r = -0.50, p = 0.005) in the left hemisphere lesion group. In the right hemisphere lesion group, the KPS score was not associated with any disconnection ratio of the right white matter tracts. The results of correlation were corrected for Bonferroni for multiple comparisons (alpha level, p = 0.0002), but these results did not withstand the correction.

### Association between white matter tract and premorbid IQ on postoperative cognitive performance

In the hierarchical multiple regression analysis for the left hemisphere lesion group, we entered age as an independent variable in Step 1 (forced entry method). The ratio of disconnection of the left AF anterior, long, and posterior segments showed significant associations in the correlation analysis and were entered as independent variables in Step 2 (stepwise method). JART scores and years of education were entered as independent variables in Step 3 (stepwise method).

The results showed that age in Step 1 did not affect the digit span forward score (β = -0.37, p = 0.06). The regression model that included only age was not significant for the digit span forward score (adjusted R^2^ = 0.10, p = 0.06). The ratio of disconnection of the left AF posterior segment, in particular, predicted the digit span forward score among the AF segments entered in Step 2 (β = -0.42, p = 0.02). The regression model that included age and the left AF posterior segment as independent variables was significant for the digit span forward score (adjusted R^2^ = 0.26, p = 0.02). Furthermore, the regression model including age and damage to the left AF posterior segment showed significantly higher explanatory power for the digit span forward score compared with the regression model with age alone as independent variable (R^2^ change = 0.18, F change = 6.13, p = 0.02). Cognitive reserve proxies entered in Step 3, such as JART and years of education, did not significantly affect the digit span forward score. Therefore, the regression model including JART or years of education set in Step 3 was not generated in the analysis of the left hemisphere patient group. The hierarchical regression analysis of this group generated only two regression models, one with only age as the independent variable and the other with age and left posterior AF as independent variables. Statistical tests were not conducted to determine whether the adding of JART or years of education as independent variables significantly increased explanatory power for the dependent variable since regression model was not generated that included JART or years of education as independent variables. Additional analysis of the interaction revealed a non-significant interaction between JART and left posterior AF disconnection (JART × left posterior AF; β = -0.01, p = 0.96).

For the right hemisphere lesion group, we entered the following independent variables in the corresponding steps: age, in Step 1 (forced entry method); ratio of disconnection of the right cingulum posterior, which showed significant associations in the correlation analysis, in Step 2 (forced entry method); and JART scores and years of education, in Step 3 (stepwise method). For the ratio of disconnection of the posterior right cingulum entered in Step 2, the correlation analysis showed that only the posterior right cingulum was significantly associated with VSTM. Therefore, we use the forced entry method in Step 2 instead of the stepwise method.

The results showed that age in Step 1 was not significantly associated with the digit span forward score (β = -0.36, p = 0.06). The regression model that included only age as an independent variable was not significant (adjusted R^2^ = 0.1, p = 0.06). Next, the ratio of disconnection of the right posterior cingulum entered in Step 2 significantly affected digit span forward scores (β = -0.46, p = 0.01). The regression model with damage to the right posterior cingulum added as an independent variable showed a significant R^2^ change compared with the regression model including only age (R^2^ change = 0.18, F change = 6.95, p = 0.01). Additionally, JART entered in Step 3 was significantly associated with digit span forward scores (β = 0.35, p = 0.03). The R^2^ change was significant, indicating that the regression model including age, JART, and damage to the right posterior cingulum significantly explained the digit span forward score better than the regression model including age and damage to the right posterior cingulum as independent variables (R^2^ change = 0.12, F change = 5.05, p = 0.03). The result of the interaction test revealed that a non-significant interaction of JART score with damage to the right posterior cingulum (JART × right cingulum posterior; β = -0.20, p = 0.22). However, a significant positive influence of JART (β = 0.40, p = 0.02) and a significant negative influence of disconnection of the right posterior cingulum (β = -0.44, p = 0.013) on digit span forward score were observed. More details are provided in Tables 2 and 3.

**Table 2:** The regression model of postoperative VSTM.

**Left hemisphere group**	**β**	**T value**	**P value**	**VIF**	**F value**	**R** ^**2**^	**Adj R** ^**2**^	**R change**	**F change**	**Sig. F change**
**Step 1**					4.02	0.14	0.10			
**Age**	-0.37	-2.01	0.06	1.00						
**Step 2**					5.49	0.31	0.26	0.18	6.13	0.02
**Age**	-0.36	-2.15	0.04	1.00						
**Left AF anterior** ^**a**^	-0.23	-0.99	0.33	1.81						
**Left AF long** ^**a**^	-0.15	-0.50	0.62	3.27						
**Left AF posterior**	-0.42	-2.48	0.02	1.00						
**Right hemisphere group**	**β**	**T**	**P**	**VIF**	**F**	**R** ^**2**^	**Adj R** ^**2**^	**R change**	**F change**	**Sig. F change**
**Step 1**					3.96	0.13	0.1			
**Age**	-0.36	-1.99	0.06	1.00						
**Step 2**					5.89	0.31	0.26	0.18	6.95	0.01
**Age**	-0.2	-1.15	0.26	1.14						
**Right cingulum posterior**	-0.46	-2.64	0.01	1.14						
**Step 3**					6.23	0.43	0.36	0.12	5.05	0.03
**Age**	-0.16	-0.99	0.33	1.15						
**Right cingulum posterior**	-0.43	-2.65	0.01	1.14						
**JART**	0.35	2.25	0.03	1.03						
**Education** ^**a**^	0.27	1.8	0.08	1.1						

VSTM, Verbal short-term memory; Left AF posterior, disconnection ratio of the left arcuate fasciculus posterior segment; right cingulum posterior, disconnection ratio of the right cingulum posterior part; JART, Japanese Adult Reading Test; Education, years of education; β, standardized partial regression coefficient; VFI, Variance Inflation Factor; R^2^, multiple correlation coefficient; Adj R^2^, Adjusted multiple correlation coefficient; Sig. F change, significance of an R^2^ change; ^a^, variables excluded from the regression model due to non-significance.

**Table 3:** The interaction effects on postoperative VSTM.

Left hemisphere group	β	T value	P value	VIF	F value	R^2^	Adj R^2^	P value
					2.63	0.32	0.2	0.062
**Age**	-0.33	-1.74	0.10	1.20				
**JART**	0.11	0.55	0.59	1.19				
**Left AF posterior**	-0.40	-2.13	0.05	1.14				
**JART×Left AF posterior**	-0.01	-0.06	0.96	1.21				
**Right hemisphere group**					5.02	0.46	0.37	0.004
**Age**	-0.13	-0.81	0.42	1.18				
**JART**	0.40	2.48	0.02	1.12				
**Right Cingulum posterior**	-0.44	-2.70	0.01	1.14				
**JART×Cingulum posterior**	-0.18	-1.11	0.28	1.11				

VSTM, Verbal short-term memory; Left AF posterior, disconnection ratio of the left arcuate fasciculus posterior segment; right cingulum posterior, disconnection ratio of the right cingulum posterior part; JART, Japanese Adult Reading Test; β, standardized partial regression coefficient; VFI, Variance Inflation Factor; R^2^, multiple correlation coefficient; Adj R^2^, Adjusted multiple correlation coefficient.

### Impact on the functional outcome

Path analysis in patients with the left hemisphere investigated the relationship as follows: (1) whether premorbid IQ and disconnection of the left AF posterior segment affect the digit span forward, digit span backward, MMSE, and KPS respectively as direct pattern; (2) whether the disconnection ratio of the left AF long segment has a negative impact on the digit span backward scores since partial correlation analysis showed that the higher disconnection ratio of the left AF long segment was correlated with lower digit span backward scores; and (3) whether premorbid IQ and disconnection of the left AF posterior segment affect KPS through digit span forward, digit span backward, and MMSE as indirect pattern. We added an error covariance between the left AF posterior segment and the long segment because it was assumed that there was a high correlation between the disconnection ratio of the left AF long segment and the left AF posterior segment.

Path analysis in patients with the right hemisphere examined as follows: (1) the same as path analysis in the left hemisphere lesion group; whether premorbid IQ and disconnection of the right cingulum posterior affect the digit span forward, digit span backward, MMSE, and KPS respectively as direct pattern; (2) the same as the (3) for path analysis in the left hemisphere lesion group; whether premorbid IQ and disconnection to the right cingulum posterior affect KPS through digit span forward, digit span backward, and MMSE as indirect pattern.

**Fig 2:**
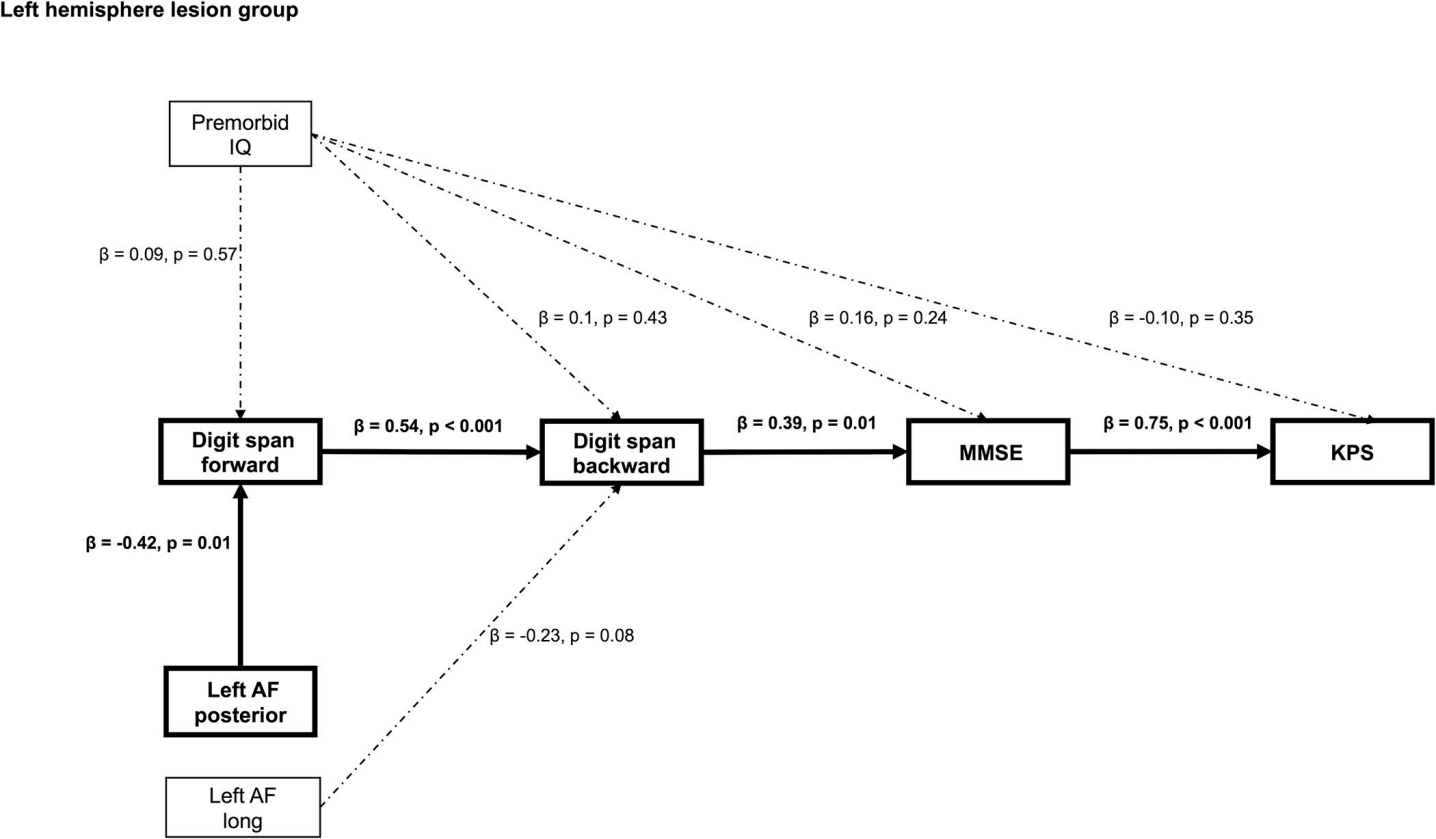
Path analysis of patients in the left hemisphere lesion group. The results of the path analysis show that damage to the left AF posterior segment has a negative impact on the postoperative performance of the digit span forward. Additionally, damage to the left AF posterior segment affects KPS through the performance of the digit span forward, digit span backward, and MMSE. Note: Left AF posterior: disconnection ratio of the left AF fasciculus posterior segment; Left AF long: disconnection ratio of the left AF fasciculus long segment; MMSE, Mini-Mental State Examination; KPS, Karnofsky Performance Status; solid line, statistically significant; dotted line, not statistically significant. We omitted the influence of age on the digit span (forward and backward), MMSE, and KPS.

Fig 2 shows the results of path analysis in the left hemisphere lesion group. For the left hemisphere lesion group, the disconnection ratio of the left AF posterior segment was significantly associated with digit span forward scores (β = -0.42, p = 0.01). The digit span forward scores significantly affected the digit span backward score (β = 0.54, p < 0.001), the digit span backward score affected the MMSE score (β = 0.39, p = 0.01), the MMSE score affected the KPS score (β = 0.75, p < 0.001). This model had a good fit (CMIN = 11.08, p = 0.79, CFI = 1.00, RMSEA = 0.00). The JART scores were not associated with the digit span scores (forward and backward), MMSE, and KPS. The disconnection of the left AF long segment did not affect the digit span backward scores. Age was associated with digit span forward scores (β = -0.35, p = 0.03) and MMSE scores (β = -0.44, p = 0.002). Age was not related to the digit span backward scores (β = -0.25, p = 0.06) and KPS scores (β = -0.16, p = 0.23).

**Fig 3:**
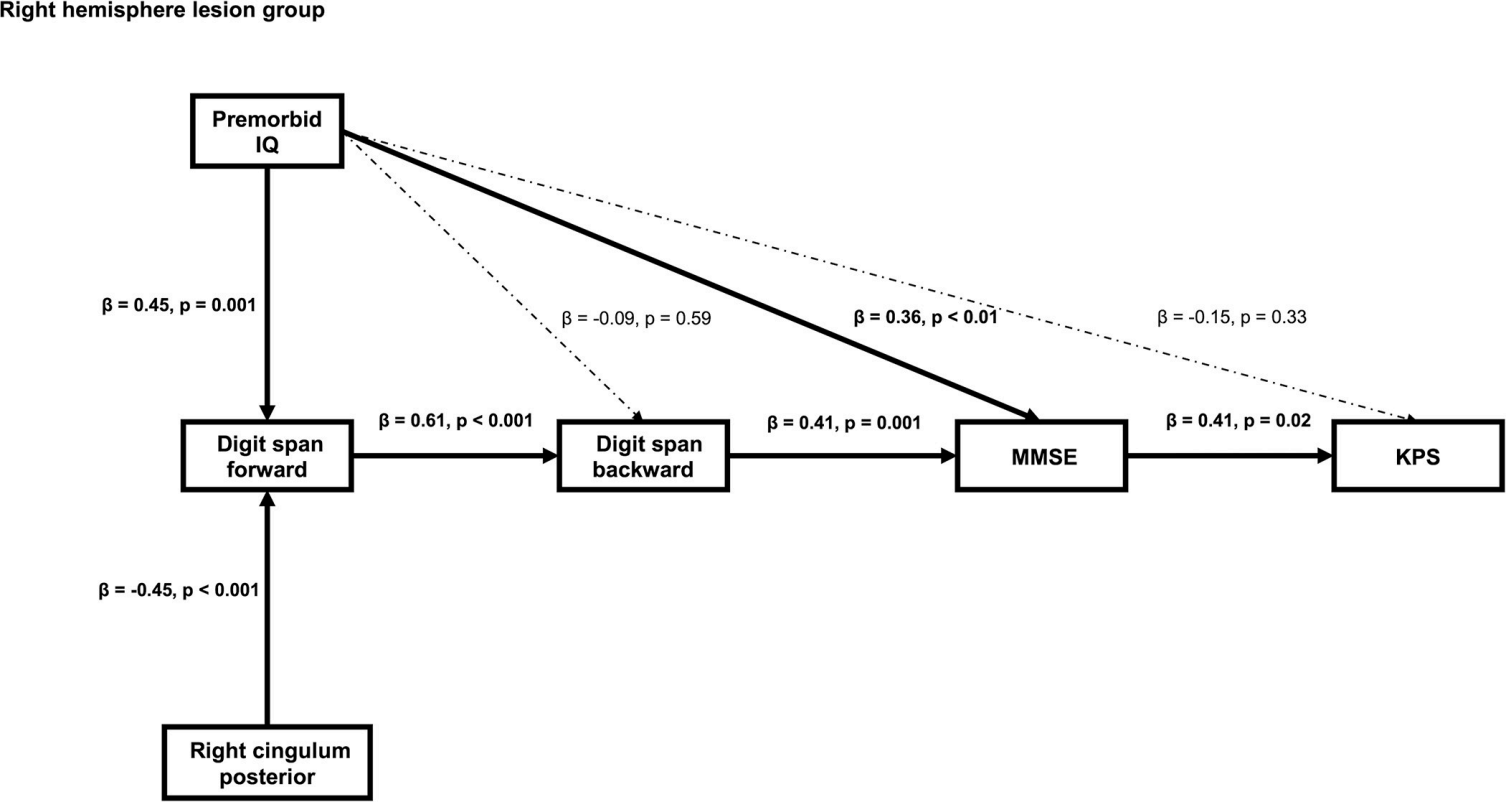
Path analysis of patients in the right hemisphere lesion group. The result of the path analysis shows a positive effect on premorbid IQ and a negative effect on damage to the right cingulum posterior part of the postoperative digit span forward. Premorbid IQ directly affects postoperative MMSE scores. Premorbid IQ and damage to the right cingulum posterior partly influence the KPS by mediating the digit span forward, digit span backward, and MMSE. Note: Right cingulum posterior: disconnection ratio of the right cingulum posterior part; MMSE, Mini-Mental State Examination; KPS, Karnofsky Performance Status; solid line, statistically significant; dotted line, not statistically significant. We omitted the influence of the age on the digit span (forward and backward), MMSE, and KPS.

Fig 3 shows the results of path analysis in the right hemisphere lesion group. For the right hemisphere lesion group, the results showed that the JART score (β = 0.45, p = 0.001) and disconnection ratio of the right cingulum posterior (β = -0.45, p < 0.001) significantly affected the digit span forward scores. The digit span forward scores significantly affected the digit span backward scores (β = 0.61, p < 0.001). JART score (β = 0.36, p = 0.004) and the digit span backward score (β = 0.41, p = 0.001) affected MMSE scores. MMSE scores significantly affected KPS scores (β = 0.41, p = 0.02). Age significantly affected MMSE scores (β = -0.36, p = 0.004) and KPS scores (β = -0.39, p = 0.01). Age was not related to the digit span forward (β = -0.16, p = 0.24) and backward scores (β = -0.09, p = 0.54). This model had a good fit (CMIN = 8.60, p = 0.48, CFI = 1.00, RMSEA = 0.00).

## Discussion

The present study investigated the influence of damage to white matter tracts and premorbid intellectual ability on postoperative VSTM and functional outcomes in patients with brain tumors. Our findings showed that damage to the left AF negatively affected postoperative VSTM and functional ability through working memory and global cognition in patients with left hemisphere lesions, whereas premorbid intellectual ability had beneficial effects on postoperative VSTM and functional ability through working memory and global cognition in patients with right hemisphere lesions. Our hypothesis was partially supported. To our knowledge, this is the first study to reveal the relationship between premorbid intellectual ability, postoperative cognitive function, and functional outcome, considering damage to the white matter tracts in patients with brain lesions.

We found that the performance of verbal working memory measured by digit span backward and verbal fluence measured by VFT in patients with brain lesions were lower than in the healthy control group. These findings suggest that patients have cognitive impairment in working memory and verbal fluency. Meanwhile, our data showed that the performance of the patients with VSTM was not lower than that of the healthy controls. These findings suggest that the patients who participated in this study did not have severe VSTM impairment that would have seriously interfered with the performance of neuropsychological test batteries. The mean MMSE score of the patients in the study was 26.8, and the mean percentage of correct answers to the naming task as one of the aphasia tests was 93.7%.

We found that severe damage to the left AF segment was associated with lower postoperative VSTM scores in patients with left hemisphere lesions. These findings are consistent with those of previous studies that found that AF is a crucial white matter tract involving VSTM [47]. Additionally, our results indicated that a higher ratio of disconnection of the posterior part of the right cingulum was correlated with VSTM in patients with right hemispheric lesions. This result is also consistent with a previous study reporting that the posterior part of the right cingulum is involved in attention [60]. Therefore, the impairment of attention due to damage to the posterior part of the right cingulum may affect VSTM, as VSTM is one of the working memory systems that is related to attention [61]. However, our results of correlation did not withstand the correction for multiple comparisons, further study with larger sample sizes are needed.

We found that the posterior part of the left AF was the best predictor of VSTM among the three AF segments in patients with left hemisphere lesions. This result suggests that damage to the left AF explains postoperative VSTM rather than other factors, such as age or proxy of cognitive reserve such as premorbid IQ and education level, in patients with left hemisphere lesions. For patients with right hemisphere lesions, premorbid IQ as cognitive reserve proxy and the damage to the right cingulum explained the postoperative VSTM. Additionally, we found that the variance of postoperative VSTM can be better explained by considering not only damage to the right cingulum but also premorbid IQ. In patients with right hemispheric tumors with an undamaged left AF, our results suggest that considering premorbid IQ level in addition to damage to white matter tracts may be more accurate in predicting postoperative VSTM. We found no significant interaction effects of cognitive reserve proxy with white matter tract disconnection in either the right or left hemisphere lesion groups. Umarova et al., [62] identified interaction effects of education attainment and age, moderated on lesion size, on cognitive impairment and disability in patients with stroke. They showed that patients with higher educational levels showed higher cognitive status measured using Montreal Cognitive Assessment (MoCA) scores and better functional outcomes measured via modified Rankin Scale (mRS) and National Institutes of Health Stroke Scale scores (NIHSS). The MoCA is used to assess cognitive impairment including a wider range of cognitive domains such as memory, visuospatial abilities, executive function, working memory, language, and orientation [63]. The mRS and NIHSS are measures that assess a broad range of functional abilities related to daily life, including physical movement, awareness, and attention. In contrast to global cognition measured using MoCA, VSTM, which we measured by digit span in our study, is a basic cognitive function that constitutes global cognition. The difference between our findings and those of previous studies suggest that the effect of premorbid IQ as a moderator variable on basic cognitive functions, such as VSTM, is not necessarily strong.

Our results showed that education level was not associated with postoperative VSTM. This result was inconsistent with a previous study showing that a higher level of education had a positive effect on post-stroke cognitive impairment [21]. One possible explanation for this result could be the overall higher educational history of the patients who participated in this study (i.e., the average of education years: minimum 12 to maximum 16 years). However, the educational level of the participants in this study was not significantly higher than that of the general Japanese population since a survey by the Japanese government has shown that the high school entrance rate in Japan was approximately 100%, and the university entrance rate was approximately 60% [64]. More research is needed in patients with a wider range of educational levels to determine whether the educational level as a proxy of cognitive reserve has a positive impact on postoperative cognitive performance.

We found that damage to the left AF is associated with VSTM directly and that damage to the left AF affects postoperative functional ability through VSTM, working memory, and global cognition indirectly in patients with left hemisphere lesions. Premorbid IQ did not affect postoperative VSTM, verbal working memory, global cognition, or functional abilities. As for the relationship between working memory, VSTM, and functional ability in the left hemisphere lesion group, our finding is consistent with previous studies that reported that VSTM and working memory were associated with functional outcomes in physical and mental-related QOL and daily living function [65–67]. In contrast to the left hemisphere lesion group, we found that damage to the posterior part of the right cingulum and premorbid intellectual ability directly impacted postoperative VSTM. Furthermore, path analysis results revealed that both factors related to VSTM affect postoperative functional ability through VSTM, verbal working memory, and global cognitive performance indirectly. The results revealed that damage to the right cingulum negatively impacted postoperative VSTM, whereas higher premorbid intellectual ability had a positive impact on postoperative VSTM. These findings suggest that premorbid intellectual ability could have a protective effect on not only postoperative VSTM but also functional ability through VSTM, working memory, and global cognition, considering the damage to the right white matter tracts associated with postoperative VSTM. This finding in right hemispheric lesions is consistent with a previous study showing that premorbid IQ is associated with language function in patients with brain tumors [29]. These findings suggest that intraoperative preservation of the left AF is critical for maintaining postoperative functional outcomes in patients with left hemisphere lesions, as damage to the left AF affects postoperative VSTM and functional ability regardless of the level of premorbid intellectual ability. In contrast, in patients with right hemispheric lesions, a higher level of premorbid intellectual ability is expected to benefit the postoperative functional prognosis, even if there is damage to the right white matter tract.

We found that functional ability was also associated with the left white matter tracts other than the AF, such as the left anterior commissure and frontal insular tract. However, we did not consider the damage to the tracts other than AF in the path analysis because our main objective was to identify the relationship between damage to AF and premorbid intellectual ability on postoperative VSTM and functional ability in patients with brain tumors. Furthermore, it was appropriate to focus on the left AF among the many white matter tracts, because a relatively large number of patients in this study had lesions in the left temporal area. Owing to the complexity of the white matter network structure and brain function, it is impossible to consider the damage to all white matter tracts in a single study. Further comprehensive studies including damage to various tracts and cognitive domains are needed to demonstrate a model that more accurately predicts postoperative functional outcomes.

This study had several limitations. First, the sample size was relatively small and the statistical power was low. Further investigation with a larger sample size is required. Second, this study included patients with stable postoperative clinical conditions who could receive a battery of cognitive function assessments. Therefore, more studies are needed to confirm whether our findings can be adapted to patients with more severe levels of postoperative cognitive impairment. Third, in a relatively large number of patients, the lesions were located in the left temporal lobe. We recommend that further studies focus on participants whose lesions are concentrated in a single location. This study focused on postoperative VSTM; we did not investigate other cognitive domains such as attention, episodic memory, and executive function. Therefore, more studies that focus on other cognitive domains are required. Fourth, we did not investigate other cognitive reserve proxies such as occupational complexity and leisure time experience. Furthermore, comprehensive research is needed to identify the effects of other cognitive reserve proxies. Fifth, patients who participated in this study had various types of tumors. Patients included those with tumor types that were not necessarily associated with neuroplasticity, such as schwannomas or hemangioblastomas. Further studies are required to demonstrate the relationship between neuroplasticity and cognitive reserve, taking into account tumor characteristics. Sixth, this is a cross-sectional study. Therefore, further studies focusing on changes in cognitive performance from preoperative to postoperative conditions are required to clarify the effects of cognitive reserve in patients with brain lesions. Finally, the method we used to measure the probability of white matter tract disconnection was an indirect approach that assesses damage by overlaying an atlas with a patient’s lesion map. Individual and group differences may occur since the atlas was obtained from a different healthy sample of participants. Further studies using diffusion data as a direct measure are needed to confirm the robustness of our results.

## Conclusion

We found that damage to the crucial white matter tract for VSTM, such as AF, affected postoperative VSTM and functional outcomes regardless of the level of premorbid intellectual ability. This finding suggests that the extent of preservation of the white matter tract in tumor resection surgery is an important factor in determining the postoperative functional prognosis. Meanwhile, if the location of the primary lesion is not in an eloquent region for VSTM, our results suggest that premorbid IQ is a predictor of postoperative VSTM and functional prognosis, as well as damage to white matter tracts. Our results highlight that if the main damage to the white matter tract is not in the eloquent region, a higher level of premorbid intellectual ability may have a beneficial effect on the postoperative functional prognosis. Therefore, in brain tumor resection surgery, in which the balance between survival prognosis and functional prognosis is important, the level of premorbid intellectual ability may be considered, as well as the extent of resection and preservation of white matter tracts, for a better prognosis.
